# Baseline Characteristics of Participants in a Randomized Controlled Trial of Metformin for Frailty Prevention

**DOI:** 10.1093/geroni/igaa057.428

**Published:** 2020-12-16

**Authors:** Tiffany Cortes, Nicolas Musi, Chen-pin Wang, Joel Michalek, Sara Espinoza

**Affiliations:** 1 University of Texas HSC at San Antonio, San Antonio, Texas, United States; 2 UTHSCSA, San Antonio, Texas, United States; 3 UT Health San Antonio, san antonio, Texas, United States; 4 UT Health Science Center San Antonio, San Antonio, Texas, United States; 5 University of Texas Health Science Center at San Antonio, San Antonio, Texas, United States

## Abstract

We are conducting a double-blind, randomized controlled trial of metformin for frailty prevention. Participants are adults aged 65+ years with pre-diabetes assessed by 2-hour oral glucose tolerance test (OGTT). Those who are frail (Fried criteria) are excluded. Participants are randomized to metformin (maximum dose of 2,000 mg/day) vs. placebo and followed for 2 years. The primary outcome is frailty (category and score); secondary outcomes are physical performance and function (short physical performance battery, 6-minute walk, lower extremity strength), systemic and skeletal muscle tissue inflammation, muscle insulin signaling, insulin sensitivity (insulin clamp), glucose tolerance (OGTT), and body composition (dual-energy x-ray absorptiometry). Safety assessments occur every 3 months; frailty, systemic inflammation, and OGTT are assessed at baseline and every 6 months, and insulin clamp with muscle biopsies are assessed at baseline and every 12 months. To date, 85 subjects have been randomized; 120 completers are planned. Mean age is 72.8 ± 5.7 years, 55.3% are male, and 43.5% were Hispanic. Mean BMI is 30.2±5.8 kg/m2, waist circumference is 104.4 ±15.5 cm, fasting glucose is 102.3 ± 10.0 mg/dL, Hemoglobin A1c is 5.8 ±0.3, and glucose at 2 hours during OGTT is 167.3 ± 17.8 mg/dL. Metformin is being examined in this study as a potential therapeutic agent to prevent frailty in older adults with pre-diabetes. Findings from this trial may have future implications for the screening and potential treatment of pre-diabetes in older patients with metformin for the prevention of frailty.

